# Effect of the SARS-CoV-2 Pandemic on Technology Use: In-Person versus Video-Chat Communication

**DOI:** 10.1093/geroni/igab046.3343

**Published:** 2021-12-17

**Authors:** Sarah Hubner, Alex Swanson, Akankshya Chataut, Stephen Kotopka, Natalie Manley, Marcia Shade, Julie Blaskewicz Boron

**Affiliations:** 1 University of Nebraska Omaha, Omaha, Nebraska, United States; 2 University of Nebraska at Omaha, Omaha, Nebraska, United States; 3 University of Nebraska Medical Center, Omaha, Nebraska, United States

## Abstract

COVID-19 risk-reduction efforts have protected high-risk individuals (including older adults) but have significantly altered life; persons now face reduced socialization. Advancing technologies (e.g., video-chat) may be useful in alleviating consequences of risk-reduction efforts, including loneliness, by improving access to alternative connection/communication across the lifespan. The purpose of this study was to investigate the relationships between technology use and individuals as this may contribute to well-being among older adults during COVID-19 and future isolating events. Participants (N=652) aged 19+ completed a questionnaire via Amazon Mechanical Turk; demographic, socialization, and technology-use data were collected. Respondents (MAge=45.15±15.81) were generally male (50.1%) and white (77.3%). In-person communication and video-chat were analyzed descriptively and with binary regressions. Results of a Wilcoxon Signed-Ranks Test indicated that video-chat (mean rank=228.45) was reported at higher frequency of use versus in-person conversations (mean-rank=202.48), Z=-4.8,p<.001). Additionally, being female positively predicted use of video chat (B=0.42,p<.05) while increasing age negatively predicted use (B=-0.01,p<.05). Regression results suggest that populations reporting higher video-chat communication (e.g., females, younger adults) may be motivated by maintaining social connectedness despite distancing and/or are committed to healthy behaviors, increasing aversion to in-person experiences. In contrast, it may be that persons reporting low video-chat use (e.g., males, older adults) may be less interested in distanced communication or may have lower technology comfort/access. Notably, sampling bias may influence results as data was collected online; future investigation is warranted. Ultimately, understanding interest in and barriers to using technology is vital to developing systems/services which support connection/communication when in-person contact is limited.

